# Osteopontin is a prognostic biomarker in non-small cell lung cancer

**DOI:** 10.1186/1471-2407-13-540

**Published:** 2013-11-11

**Authors:** Ane Kongsgaard, Kjetil Boye, Miriam Øijordsbakken, Marius Lund-Iversen, Ann Rita Halvorsen, Steinar K Solberg, Gisle Berge, Åslaug Helland, Odd Terje Brustugun, Gunhild M Mælandsmo

**Affiliations:** 1 Department of Tumor Biology, Institute for Cancer Research, The Norwegian Radium Hospital, Oslo University Hospital, Oslo, Norway; 2 Department of Oncology, The Norwegian Radium Hospital, Oslo University Hospital, Oslo, Norway; 3 Department of Medical Biochemistry, The Norwegian Radium Hospital, Oslo University Hospital, Oslo, Norway; 4 Department of Pathology, The Norwegian Radium Hospital, Oslo University Hospital, Oslo, Norway; 5 Department of Genetics, Institute for Cancer Research, The Norwegian Radium, Hospital, Oslo University Hospital, Oslo, Norway; 6 Department of Cardiovascular and Thoracic Surgery, Rikshospitalet, Oslo University Hospital, Oslo, Norway; 7 Department of Pharmacy, Faculty of Health Sciences, University of Tromsø, Tromsø, Norway

**Keywords:** NSCLC, Prognosis, Biomarker, Osteopontin, S100A4

## Abstract

**Background:**

In a previously published report we characterized the expression of the metastasis-associated proteins S100A4, osteopontin (OPN) and ephrin-A1 in a prospectively collected panel of non-small cell lung cancer (NSCLC) tumors. The aim of the present follow-up study was to investigate the prognostic impact of these potential biomarkers in the same patient cohort. In addition, circulating serum levels of OPN were measured and single nucleotide polymorphisms (SNP) in the -443 position of the OPN promoter were analyzed.

**Methods:**

Associations between immunohistochemical expression of S100A4, OPN and ephrin-A1 and relapse free and overall survival were examined using univariate and multivariate analyses. Serum OPN was measured by ELISA, polymorphisms in the -443 position of the tumor OPN promoter were analyzed by PCR, and associations between OPN levels and promoter polymorphisms and clinicopathological parameters and patient outcome were investigated.

**Results:**

High expression of OPN in NSCLC tumors was associated with poor patient outcome, and OPN was a strong, independent prognostic factor for both relapse free and overall survival. Serum OPN levels increased according to tumor pT classification and tumor size, and patients with OPN-expressing tumors had higher serum levels than patients with OPN-negative tumors. S100A4 was a negative prognostic factor in several subgroups of adenocarcinoma patients, but not in the overall patient cohort. There was no association between ephrin-A1 expression and patient outcome.

**Conclusions:**

OPN is a promising prognostic biomarker in NSCLC, and should be further explored in the selection of patients for adjuvant treatment following surgical resection.

## Background

Lung cancer, with non-small cell lung cancer (NSCLC) constituting 85% of cases, retains its position as one of the most commonly diagnosed cancer forms globally. In fact, lung cancer is the leading cause of cancer death in men, and the second leading cause of cancer death in women [1]. The overall 5-year survival rate for NSCLC is poor, and even for patients with early stage disease who undergo curatively intended surgery, the post operative recurrence rate is high compared to other types of cancer [2]. Nearly half of NSCLC patients undergoing surgical resection experience disease relapse, and in these patients disease stage according to TNM, followed by age and gender, are the most important prognostic factors [3]. However, even in patients with early stage NSCLC there are substantial differences in recurrence rates, reflecting the biological heterogeneity and complexity of these tumors. As the current TNM staging does not provide satisfactory prognostication of the patients, it is essential to identify novel prognostic biomarkers and determine if they are applicable in the subclassification of patients. Also, novel therapies in NSCLC are certainly warranted, and as targeted treatment is becoming increasingly important, identifying molecular markers as potential therapeutic targets is necessary.

In a prospectively collected panel of tumor tissue from 244 NSCLC patients undergoing curatively intended surgery, we have previously characterized the expression of the metastasis-associated proteins S100A4, osteopontin (OPN) and ephrin-A1, and investigated the associations between these proteins and clinical and histopathological parameters [4]. S100A4, a member of the S100 protein family, is involved in several steps of the metastatic cascade and is associated with patient outcome in various types of cancer [5]. In NSCLC, several studies have shown S100A4 to be related to poor prognosis [6-8], whereas others have reported no association between S100A4 and patient outcome [9,10]. OPN, a multifunctional protein secreted by a variety of cells [11], is associated with cancer development, progression and metastasis in different malignancies, including NSCLC [12-17]. Circulating plasma OPN levels in NSCLC patients have been shown to correlate with disease stage [16,18] and with survival [19,20]. OPN can undergo extensive post-translational modifications and alternative RNA splicing [11], and polymorphisms in the OPN promoter have been shown to affect its transcriptional activity [18,21].

Being a ligand for several of the Eph family receptor tyrosine kinases, the cell surface protein ephrin-A1 is involved in multiple biological processes including metastasis and tumor angiogenesis [22]. In lung cancer however, results are conflicting, as high expression has also been associated with favorable prognostic factors in NSCLC [2] and improved overall survival in lung adenocarcinoma [23].

In our previously published report we showed that S100A4, OPN and ephrin-A1 were highly expressed in NSCLC tumor tissue, and that S100A4 expression was associated with adenocarcinoma histology, as well as with small tumor size and high degree of differentiation. S100A4, OPN and ephrin-A1 are all potentially interesting biomarkers that may have clinical impact in NSCLC, and in this follow-up study we investigate the association between the expression of these proteins and patient outcome in the previously described cohort. In addition, pre-surgery serum OPN levels were measured and single nucleotide polymorphisms (SNP) in the -443 position of the OPN promoter were analyzed. The potential relationships between OPN promoter polymorphism, expression in primary tumor biopsies and circulating levels of OPN were investigated, and assessed in relation to patient outcome.

## Methods

### Patient cohort

Between March 2006 and April 2010, primary tumor samples were prospectively collected from 244 patients with assumed or verified NSCLC who underwent curatively intended surgical resection at Rikshospitalet, Oslo University Hospital, Oslo, Norway. The study was approved by the Regional Ethics Committee (S-06402b), and written informed consent was obtained from all patients. Resected tissue was processed for routine histopathological assessment, and histological examination of all tissue specimens was performed by an experienced pathologist. Tumors were staged according to the International Association for the Study of Lung Cancer (IASLC), TNM 7, and the histological subtypes were classified according to WHO criteria. Thirty-four patients were excluded from the statistical analyses for the following reasons: histology other than NSCLC (carcinoid (12), small cell lung cancer (4), lung metastases from other primary cancer (7)), metastatic disease at the time of surgery (3), inadequate surgical margins (4), and withdrawal of consent (4). The study population thus included 210 patients with histologically verified primary NSCLC in pTNM stage I-III who had undergone curatively intended surgery. Postoperatively, patients were followed by clinical evaluation and radiological examination (CT or conventional x-ray of the chest) in their respective local hospitals according to national guidelines. Follow-up data were obtained from these hospitals and by contacting the patient’s general practitioner. In addition, survival data were obtained from the National Registry of Norway and updated on August 14th 2012.

### Tissue microarray (TMA) construction and immunohistochemistry

The tissue microarray construction and the immunohistochemical staining procedures have been described in detail previously [4]. Briefly, the most representative tumor areas in each tumor tissue donor block were selected and marked on hematoxylin-eosin stained sections, and at least two cores from different tumor areas of the same specimen were included in the TMA. TMA sections were constructed using a tissue arrayer instrument (Beecher Instruments, Silver Springs, MD, USA). Immunohistochemical staining was done using the EnVision™ FLEX + detection system (Dako, Glostrup, Denmark) for S100A4 and OPN, and the EnVision + system (Dako) for ephrin-A1. The following primary antibodies were used: mouse monoclonal anti-S100A4 (20.1) [24], final concentration 3 μg/ml, rabbit polyclonal anti-osteopontin 0.67 μg/ml (Rb-9097, Thermo Fisher Scientific, Fremont, CA, USA) and rabbit polyclonal anti-ephrin-A1 0.67 μg/ml (sc-911, Santa Cruz Biotechnology, Santa Cruz, CA, USA). For positive controls, sections from colorectal tumor tissue, ovarian tissue and cervical portio biopsy tissue known to express high amounts of S100A4, OPN and ephrin-A1, respectively, were used. Information regarding the evaluation of the immunohistochemical staining has been reported in Rud *et al.*[4]. In brief, for S100A4 cytoplasmic and nuclear immunoreactivity was recorded. The samples were scored using a 0–3 scale according to staining intensity, with 0 denoting negative (no staining), 1 denoting weak staining, 2 intermediate staining and 3 strong staining. For nuclear staining, the fraction of positively stained nuclei was estimated (0 = 0%, 1 = < 1%, 2 = 1 – 10%, 3 = 11 – 33%, 4 = 34 – 66% and 5 = 67 – 100%). All samples with >10% stained nuclei (score ≥ 3) were considered positive, and grouped according to staining intensity. The same tumors had strong S100A4 staining in the cytoplasm and in the nucleus, and for that reason data analyses for nuclear S100A4 staining individually did not provide further information. The negative and weakly stained S100A4 cases were pooled into one group for the statistical analyses. OPN and ephrin-A1 cytoplasmic immunoreactivity was scored according to a 0–2 scale, with 0 defined as negative (no staining), 1 as intermediate staining and 2 as strong staining.

### Measurement of serum OPN concentration

The OPN levels in serum were measured with the ELISA kit Quantikine Human Osteopontin Immunoassay DOST00 from R&D (R&D Systems Inc., Minneapolis, Minnesota, USA), according to the manufacturer’s manual. In brief; serum samples from each patient were diluted 1:10 with calibrator Diluent RD5-24 and incubated in an OPN antibody-coated micro titer plate for 2 hours at room temperature. After washing the wells four times, 200 μl OPN conjugate (polyclonal antibody against OPN conjugated to horseradish peroxidase) was added to each well and incubated for 2 hours at room temperature. Following four washes, 200 μl substrate solution (hydrogen peroxide and chromogen) was added to each well and incubated for 30 minutes at room temperature. The samples were measured on a plate reader Victor 1420 Multilabel Counter, (Wallac/PerkinElmer Life Sciences, Turku, Finland) at 450 nm with wavelength correction at 570 nm. Standard curve and sample values were calculated by use of the Wallac MultiCalc program.

### Analysis of polymorphisms in the OPN promoter

For analysis of the -443 OPN promoter polymorphism (rs17730582) in primary NSCLC, total DNA was isolated from 210 tumor specimens using Maxwell 16 DNA Purification Kit (Promega, Madison, WI, USA) according to the manufacturer’s manual. A fluorescently marked fragment of the OPN gene containing the SNP rs 11730582 was amplified by PCR. The PCR reaction consisted of 50 ng genomic DNA, 0.08 U/μl *Taq*, polymerase, 0.005 U/μl PFU polymerase, 1× Buffer 4 (ABgene), 3 mM MgCl_2_, and 0.4 mM dNTP mix (ABgene) and 0.2 μM of the primer (5’-Fam-CGCCCGCCGCGCCCCGCGCCCGTCCGCCGkit Quantikine Human OCCCCCGCCCGGGAGCTTGAGTAGTAAAGGACA-3’) and 0.3 μM of the primer (5’AGAATGGTCCTGCACCAGTAA3’). Temperature cycling was performed in a DNA Engine Tetrad 2 Thermal Cycler (Biorad, CA, USA) using the following cycling conditions: denaturation 5 min at 95°C, followed by 35 cycles of 95°C in 30 s, annealing 57°C in 30s and 72°C in 60s. For variant detection amplified 6-fam labelled PCR products were analyzed by denaturant capillary electrophoresis in a MegaBACE 1000 DNA Analysis System (GE Healthcare Bio-Sciences AB, Uppsala, Sweden). The base variants were separated by cycling temperature capillary electrophoresis (CTCE), with mean separating temperatures of 52,5°C and amplitudes of 3°C cycled 20 times. The variants were identified by co-analysis with a mutated internal standard in a similar manner as previously described by Bjørheim *et al*. [25].

### Statistical analyses

Univariate survival analysis was performed according to the Kaplan-Meier method, and the log rank test was used to evaluate the statistical significance between survival curves. Multivariate survival analysis was performed using the Cox proportional hazard regression model with backward, stepwise elimination of variables. Relapse free survival was calculated from the date of surgery until the date of diagnosis of local recurrence or metastasis, or until the date of the last follow-up visit for healthy patients. Median follow-up for patients still alive who had not developed metastasis or local recurrence was 34.0 months (range 12.5 – 52.9 months). Overall survival was measured from date of surgery until date of death. Associations between OPN promoter -443 genotypic variations and immunohistochemical expression were examined using linear by linear association chi-square test. For analyses of associations between serum OPN levels and clinicopathological parameters, parametric tests (independent samples t-test or one-way ANOVA, as appropriate) were used. All data analyses were performed using SPSS statistical software version 18.0 (SPSS Inc., Chicago, IL, USA), and p- values < 0.05 were considered statistically significant.

## Results

### Patient characteristics and outcome

Clinicopathological parameters and outcome parameters of the study cohort are summarized in Table 1. The mean patient age was 65 years (range 43–82) for women and 67 years (range 39 – 83) for men. The NSCLC tumors included 61% adenocarcinomas, 28% squamous cell carcinomas and 11% large cell carcinomas. Sixty-four percent of the patients were in pTNM stage I, 21% of patients in pTNM II, and 15% in pTNM stage III. Adjuvant chemotherapy was administered in 66 cases (31%), and 5 patients (2%) received postoperative radiotherapy. Sixty four patients (31%) presented disease relapse (local recurrence or metastases) during follow-up. A total of 70 (33%) patients had died at the end of follow-up.

**Table 1:** Characteristics of patient cohort

**Parameter**		**Patients**	
		**Number**	**Percent**
Gender	Male	114	54
	Female	96	46
Histology	Adenocarcinoma (incl. BAC)	128	61
	Squamous cell carcinoma	58	28
	Large cell carcinoma	24	11
Differentiation	G1 (well differentiated)	20	10
	G2 (moderately differentiated)	135	68
	G3 (poorly differentiated)	43	22
	Missing	12	
pTNM	I	135	64
	II	43	21
	III	32	15
pT	pT1	67	32
	pT2	114	54
	pT3	19	9
	pT4	10	5
pN	0	153	73
	1	38	18
	2	19	9
Tumor size	≤ 2.0 cm	56	27
	2.1 – 3.0 cm	61	29
	3.1 – 5.0 cm	64	30
	5.1 – 7.0 cm	23	11
	> 7.0 cm	6	3
Metastasis*	Yes	55	26
	No	155	74
Local recurrence	Yes	24	11
	No	186	89
Death	Yes	70	33
	No	140	67

*Metastasis during the follow-up period.

### Associations between clinicopathological parameters and patient outcome

The prognostic significance of conventional clinicopathological variables was first investigated by univariate analysis and the results are presented in Table 2. Tumor size > 3.0 cm and poor tumor differentiation (grade 3) were significantly associated with disease relapse (p = 0.04 and 0.03, respectively) and with overall survival (p = 0.04 and 0.01, respectively). Differences in relapse free survival were observed for pTNM stage I – III and lymph node involvement status, but these were not statistically significant. Patients < 65 years had a better overall survival (p = 0.05). None of the other parameters showed a statistically significant correlation with patient outcome. Multivariate analyses were performed including the following parameters; age, gender, histology, tumor size and differentiation, and only tumor size was significantly associated with relapse free survival (p = 0.008, hazard ratio (HR) 1.2; 95% confidence interval (CI) 1.1-1.4; data not shown).

**Table 2:** Univariate survival analysis of clinicopathological parameters and OPN

	**Relapse free survival**			**Overall survival**		
**Parameter**	p*	**HR**	**95% CI**	p*	**HR**	**95% CI**
**Gender**	0.67			0.48		
Female						
Male		0.9	0.6–1.5		1.2	0.7–1.9
**Age**	0.81			0.05		
< 65 years						
> 65 years		1.1	0.6–1.7		1.6	1.0–2.7
**Histology**	0.91			0.08		
Adenocarcinoma (incl.BAC)						
Squamous cell carcinoma		1.0	0.6–1.8		1.7	1.0–2.9
Large cell carcinoma		1.2	0.6–2.4		1.6	0.8–3.1
**pTNM**	0.28			0.97		
I						
II		1.2	0.7–2.2		1.0	0.5–1.8
III		1.4	0.7–2.7		1.0	0.5–2.1
**pT**	0.26			0.74		
pT1						
pT2		1.1	0.6–2.0		1.3	0.8–2.3
pT3		1.4	0.6–3.4		0.9	0.3–2.4
pT4		1.8	0.6–5.2		1.2	0.4–4.2
**pN**	0.18			0.27		
pN0						
pN1–2		1.4	0.8–2.4		1.3	0.8–2.2
**Tumor size**	0.04			0.04		
≤ 3.0 cm						
≥ 3.1 cm		1.7	1.0–2.7		1.6	1.0–2.6
**Differentiation**	0.03			0.01		
G1 (well)						
G2 (moderate)		2.4	0.7–7–6		2.5	0.8–8.0
G3 (poor)		3.5	1.0–11.9		3.9	1.2–13.4
**Osteopontin**	0.005			0.04		
0						
1		2.0	0.9–4.5		1.2	0.6–2.4
2		3.9	1.5–10.4		2.7	1.2–6.3

*p-values calculated by log rank test.

### Associations between S100A4 and ephrin-A1 expression and patient outcome

**Figure 1:**
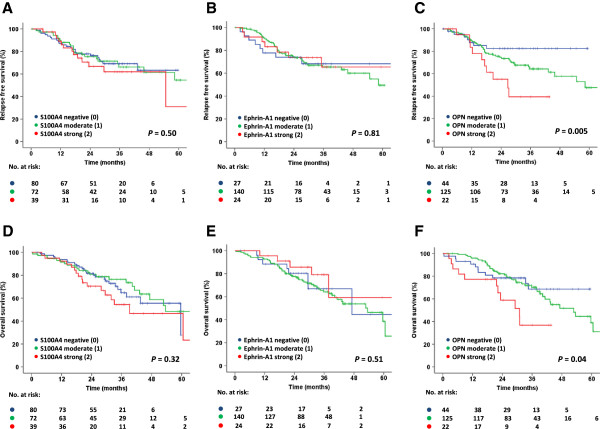
Kaplan-Meier survival plots depicting relapse free survival (A-C) and overall survival (D-F) based on immunohistochemical expression of S100A4 (A, D), ephrin-A1 (B, E) and OPN (C, F).

The immunohistohemical staining pattern and distribution of OPN, S100A4 and ephrin-A1 in the tumor tissues have been described previously [4]. Five patients from the previous cohort were not included in the present analysis, and Table 3 gives an overview of the immunohistochemical expression in the present cohort. As shown in Figure 1A, there were no significant differences in relapse free survival between patients with negative/weak, moderate and strong S100A4 expressing tumors. We found a tendency for worse overall survival in S100A4-positive patients, and to explore this further we performed statistical analyses with only two groups; tumors with strong S100A4 staining were categorized as positive and the remaining tumors were categorized as negative. S100A4-positive patients had poorer overall survival, however the difference did not reach statistical significance (p = 0.09). Similar observations were seen when we analyzed the adenocarcinoma patients as a separate group. A better, yet not statistically significant, overall survival was seen for patients with S100A4-negative tumors (p = 0.06). When patients were stratified by disease stage, S100A4 was associated with poor outcome in pTNM I (p = 0.04). S100A4 was also a negative prognostic factor in lymph node negative patients, where 3-year overall survival for patients with S100A4-positive and negative tumors was 56% and 83%, respectively (p = 0.01).

**Table 3:** **Immunohistochemical expression of OPN**, **S100A4 and ephrin**-**A1**

	**Number**	**Percent**
**OPN**		
Negative	44	23
Moderate	125	65
Strong	22	12
**S100A4**		
Negative/weak	80	42
Moderate	72	38
Strong	39	20
**Ephrin**-**A1**		
Negative	27	14
Moderate	140	73
Strong	24	13

Further subgroup analyses of the overall cohort were performed and showed that in patients with pT2 tumors, S100A4 expression was a significant negative prognostic factor for relapse free and overall survival (p = 0.03 and 0.001, respectively, data not shown). Three-year relapse free survival for S100A4-positive patients in this group was 45%, compared to 71% for S100A4-negative patients. Ephrin-A1 was not a significant prognostic marker in this patient cohort (Figure 1B and 1E) and no prognostic impact was revealed when performing subgroup analyses.

### Association between OPN expression and patient outcome

**Figure 2:**
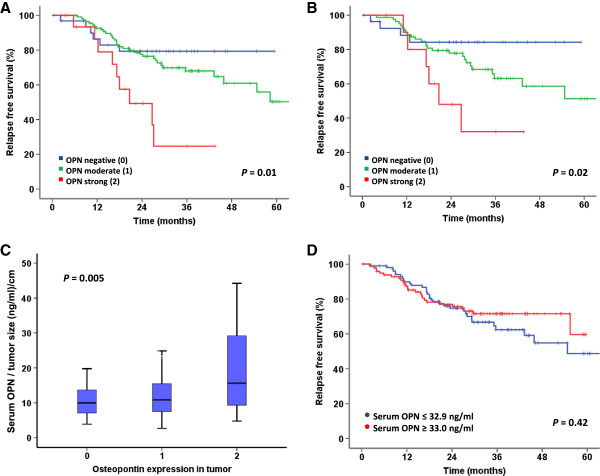
**Associations between osteopontin expression, serum concentrations and patient outcome. A** and **B**: Kaplan-Meier survival plots depicting relapse free survival based on tumor expression levels of OPN in the subgroup of patients with pTNM stage I and II **(A)** and in the subgroup of patients with adenocarcinomas **(B)**. **C**: Box plots showing relative serum OPN concentrations according to tumor OPN expression levels. Since larger tumors are expected to secrete more OPN than small tumors given the same OPN expression levels, the serum OPN concentrations have been divided by tumor diameter. Data show median values (horizontal line within the box) and interquartile range (upper and lower border of the box) of serum OPN concentrations. The upper and lower horizontal bars represent the maximum and minimum values, respectively. Outliers have been removed from the plot. **D**: Kaplan-Meier survival plot depicting relapse free survival based on serum OPN level, patients dichotomized at the median OPN level. P-values calculated by one-way ANOVA test **(C)** and log-rank test **(A, B, D)**.

Representative images of the immunohistochemical expression of OPN are shown in Additional file 1: Figure S1. The expression of OPN was strongly associated with poor relapse free survival (p = 0.005, Figure 1C and Table 2). Patients with high OPN expression in the tumor (12% of cases) had a 3-year relapse free survival rate of 39%, compared to 64% for patients with moderate OPN expression (65% of the patients) and 83% for patients whose tumors were negative for OPN. A similar trend was found for overall survival, where 3-year survival rates for high, moderate and low expression of OPN was 38%, 70% and 71%, respectively (p = 0.04, Figure 1F). Furthermore, subgroup analysis of only stage I-II patients were performed and showed that OPN expression was significantly associated with both relapse free survival (p = 0.01, Figure 2A) and overall survival (p = 0.04, data not shown). Stratification of the patients according to histological subtype revealed a prognostic impact of OPN in adenocarcinoma patients (p = 0.02, Figure 2B). To determine if the relationship between OPN expression and patient outcome was independent of other clinical and pathological parameters, multivariate analyses including the following parameters were performed: OPN, age, gender, pT category, pN category, tumor differentiation and histology. Interestingly, OPN was independently and significantly associated with both relapse free survival (p = 0.02, HR 3.9; 95% CI 1.5-10.4; for strong OPN staining compared to negative staining; data not shown) and overall survival (p = 0.05; HR 2.8; 95% CI 1.2-6.5; for strong OPN staining compared to negative staining; data not shown).

### Measurement of serum OPN concentration

Since immunohistochemical expression of OPN was associated with poor outcome, we investigated if serum levels of OPN could reflect expression in the primary tumor and be of prognostic significance. Serum samples for measurement of circulating OPN were available from 201 of the 210 patients, and OPN concentrations ranged from 8.1 to 164.1 ng/ml, with a mean level of 36.6 ng/ml and a median value of 32.9 ng/ml. OPN was also measured in serum samples from a control group of 30 healthy individuals (blood donors), where the mean level was 22.9 ng/ml and the median value 20.3 ng/ml (range 9.5 – 49.1 ng/ml). The difference in serum levels between the patients and the donors was statistically significant (p < 0.001, independent samples t-test). Associations between serum OPN concentrations and clinicopathological parameters of the patient cohort are summarized in Table 4. Circulating OPN levels were higher in male patients compared to female patients (p = 0.02). Small differences in concentration according to pTNM stage were observed, but these were not statistically significant. However, OPN levels were significantly associated with both pT classification and tumor size (p = 0.006 and 0.01, respectively; Table 4). In fact, the median OPN concentration in patients with pT1 tumors was 28.0 ng/ml, compared to 33.9 for pT2, 35.2 for pT3 and 39.6 for pT4. Among the histological subtypes, patients with large cell carcinomas and squamous cell carcinomas had higher OPN levels than adenocarcinoma patients, but the difference did not reach statistical significance (Table 4).

**Table 4:** Associations between patient characteristics by serum OPN concentration

**Parameter**	**Median serum OPN concentration (IQ Range)**	**p**-value*
**Gender**		0.02
Female	27.9 ng/ml (18.6)	
Male	35.2 ng/ml (19.5)	
**Age**		0.41
< 65 years	32.8 ng/ml (17.8)	
> 65 years	33.3 ng/ml (24.4)	
**Histology**		0.17
Adenocarcinoma	30.1 ng/ml (18.7)	
Squamous cell carcinoma	35.0 ng/ml (21.3)	
Large cell carcinoma	39.7 ng/ml (23.2)	
**pTNM**		0.42
I	31.0 ng/ml (20.7)	
II	35.8 ng /ml (16.9)	
III	35.2 ng/ml (22.2)	
**pT**		0.006
pT1	28.0 ng/ml (25.2)	
pT2	33.9 ng/ml (22.7)	
pT3	35.2 ng/ml (21.7)	
pT4	39.6 ng/ml (18.6)	
**pN**		0.79
pN0	31.8 ng/ml (22.7)	
pN1	35.4 ng/ml (15.6)	
pN2	32.9 ng/ml (26.6)	
**Tumor size**		0.01
≤ 3.0 cm	29.1 ng/ml (17.9)	
≥ 3.1 cm	36.0 ng/ml (19.3)	

*p-value calculated by one-way ANOVA test or independent samples t-test as appropriate.

No statistically significant association was found between serum OPN concentrations and OPN expression in the primary tumor (p = 0.09), but patients with high OPN expression had numerically higher serum levels than patients with OPN-negative tumors (median values of 37.0 ng/ml and 31.0 ng/ml, respectively). However, a direct comparison between serum levels and immunohistochemical expression without taking the size of the tumor into account might not be relevant, as larger tumors would be expected to secrete more OPN than small tumors given the same OPN expression levels. Tumor diameter measured in one dimension was the only parameter available describing tumor size in this study. Even though this is an imperfect surrogate of tumor volume, we found that OPN serum levels divided by tumor diameter was closely associated with OPN expression in the primary tumor. Patients with OPN-negative tumors had a relative median serum level of 9.9 ng/ml, compared to 10.8 and 15.6 ng/ml in patients with moderate and strong expression, respectively (p = 0.005, Figure 2C).

In Cox univariate analysis the total serum OPN levels were not associated with overall survival (p = 1.0; HR 1.0; 95% CI 0.99-1.01) or relapse free survival (p = 0.7; HR 0.9; 95% CI 0.9-1.0, data not shown). In addition, there was no correlation to relapse free survival (p = 0.42, Figure 2D) or overall survival (p = 0.37, data not shown) when using log-rank tests with patients dichotomized into two groups at the median serum concentration. Survival analyses were also performed with patients divided into tertiles and quartiles according to serum OPN concentration without revealing any associations with outcome.

### Analysis of polymorphisms in the -443 position of OPN promoter

Polymorphisms in the -443 position of the OPN promoter were analyzed in available tumor DNA from 174 patients in the cohort. Overall 101 samples (58%) were heterozygous (-443C/T), 40 (23%) were homozygous for CC and 33 samples (19%) were homozygous for TT. There was no association between the genotype and the level of OPN expression in the tumors (p = 0.4, data not shown) or between genotype and serum OPN concentrations (p = 0.2, data not shown). Also, OPN promoter polymorphism did not affect the outcome of the patients for either relapse free survival or overall survival (p = 0.8 and 0.9, respectively, data not shown).

## Discussion

In the present prospective study we have investigated the prognostic impact of OPN, S100A4 and ephrin-A1 in a previously described cohort of 210 surgically resected NSCLC patients [4]. We have shown that tumor OPN expression is a strong predictor of poor prognosis, and multivariate analysis confirmed OPN as an independent prognostic factor. OPN plays an important role in tumorigenesis, progression and metastatic dissemination in several cancer types including NSCLC [15], and our results are in line with previous studies on OPN expression in NSCLC [13-15,17]. The present study is strengthened by the fact that the patients have been prospectively recruited and the cohort is therefore unbiased. Furthermore, this is to our knowledge the first report investigating tumor OPN expression levels, serum levels and genotypic variations in the OPN promoter in NSCLC in the same patient cohort.

The finding that patients with OPN-expressing tumors have worse relapse free and overall survival than patients with OPN-negative tumors indicates that OPN has the potential to be used as a prognostic biomarker in NSCLC. According to national guidelines, patients in pTNM stage II or III who are under 70 years should be offered adjuvant chemotherapy following surgery. We found that in this group, OPN-negative patients showed a particularly good outcome, as their 3-year relapse free survival was similar to stage I patients below 70 years (who are not offered adjuvant chemotherapy). Also, in the latter group, the patients with OPN-positive tumors showed a particularly poor outcome. Consequently, OPN expression, if validated in future studies, could be used for selection of patients for adjuvant treatment following surgical resection of NSCLC.

Former studies have shown that also circulating OPN levels in serum or plasma are increased in patients with various forms of cancer, including NSCLC [16], and that increased level is associated with poor prognosis [19,20]. In the present patient cohort we found a median serum OPN level of 32.9 ng/ml, which is comparable to results from previous studies on OPN in serum [19] or plasma [18,26]. Serum values of OPN are known to be significantly lower than plasma values due to proteolytic cleavage by thrombin during coagulation, and in our experience the serum concentrations are approximately half of plasma concentrations. We showed that there is a relationship between serum OPN levels and tumor OPN expression, as patients with OPN-expressing tumors had higher serum levels than patients with OPN-negative tumors, and this difference became significant when tumor size was taken into account. Furthermore, this study showed that serum OPN levels increased according to pT classification and tumor size, however there was no association to patient outcome.

The finding that tumor OPN expression, but not serum OPN level, was associated with poor survival may be explained by the multi-functionality of OPN. The majority of the activities of OPN have been ascribed the interaction between secreted OPN and its receptors on target cells [27], however OPN is also found intracellularly and the nonsecreted form is involved in cellular processes such as migration and motility [27,28]. Our results may indicate that the intracellular levels of OPN are more important than the secreted circulating levels in NSCLC. Moreover, variations in serum OPN measurements may occur due to proteolytic cleavage of circulating OPN [29]. Finally, OPN has an important role in inflammation and wound healing [26], and therefore other systemic sources than the tumor itself may affect OPN levels in the circulation.

There are several polymorphic sites in the regulatory element of the OPN promoter, and SNPs at nucleotide -443 are frequently detected and reported involved in regulation of OPN expression in normal cells [18,21] as well as in cancer cells [30,31]. When analyzing the genotypic variations at position -443 in NSCLC tumor DNA we found no association to the expression levels of OPN at the protein level. The heterozygous -443 T/C was the most common variant, however in our material this polymorphism does not seem to be responsible for the different expression levels of OPN in the tumors. Notably, the literature on this matter is conflicting as it has been reported that melanoma metastases homozygous for the -443C allele expressed higher levels of OPN [31], but another study on hepatocellular carcinoma showed an association between the -443TT genotype and increased expression of OPN [30]. A recently published study on advanced NSCLC patients reported that patients with the -443CC genotype in their genomic DNA had significantly lower survival rates than patients with the two other genotypes [32].

S100A4 has been related to poor patient outcome in several cancer types [5]. In our previously published report we found that S100A4 expression was associated with smaller, highly differentiated NSCLC tumors and that S100A4 had a significantly higher expression in adenocarcinomas compared to the other histological subtypes [4]. In this group of patients, S100A4 expression was higher in pTNM stage I than in stage II - III, and in lymph node negative compared to lymph node positive patients. These results were unexpected, and could be indicative of S100A4 as a positive prognostic factor in the examined patient cohort. However, in the present follow-up study we found a tendency for shorter survival time in patients with S100A4-positive tumors, although the difference was not statistically significant. Subgroup analyses showed that S100A4 was associated with unfavourable prognosis in patients with pT2 tumors. In addition, in the adenocarcinomas, S100A4 had negative prognostic impact also in stage I and in lymph node negative patients. These results are consistent with several previous reports on S100A4 in NSCLC [6-8], and further suggest that S100A4 is a negative prognostic factor in early-stage NSCLC, and especially in lung adenocarcinoma. In the present study there was no association between ephrin-A1 expression and patient outcome. Previous studies on ephrin-A1 in NSCLC have been conflicting, as associations to improved patient outcome have been reported [2,23], while upregulation of the ephrin-A1 receptor EphA2 has also been related to poor clinical outcomes in many types of cancer [2].

pTNM stage is considered to be the most important prognostic factor in NSCLC [3], but in our cohort no statistically significant association between pTNM stage and survival was detected. The numbers of patients with stage II, and especially stage III disease, were relatively small compared to stage I, and this may have affected the statistical analysis. Also, stage III NSCLC patients represent a heterogenous group in which the optimal treatment differs according to the T and N stage. As our cohort includes only patients who were considered operable and underwent curatively intended surgery, the fact that these patients present better outcome than stage III NSCLC in general is not surprising. Subgroup analysis of stage I - II patients alone showed that OPN expression was significantly associated with both relapse free- and overall survival, indicating that OPN might be a particularly promising biomarker in early stage NSCLC.

## Conclusions

This study provides further evidence of the importance of OPN in the biology of NSCLC. OPN may have the potential to be used as a biomarker to select patients for adjuvant treatment following surgical resection, and to clarify this issue OPN expression in tumors from patients included in clinical studies on adjuvant chemotherapy should be investigated.

## Competing interests

The authors declare that they have no competing interests.

## Authors’ contributions

AK conceived the study, evaluated immunostained sections, performed data analysis and wrote the manuscript. KB conceived the study, evaluated immunostained sections, participated in data analysis and manuscript drafting. MØ and GB performed the OPN serum measurements. ML-I evaluated immunostained sections. ARH, OTB, ÅH and SKS provided patient material and patient data. GMM conceived the study and participated in writing the manuscript. All authors read and approved the final manuscript.

## Pre-publication history

The pre-publication history for this paper can be accessed here:

http://www.biomedcentral.com/1471-2407/13/540/prepub

## Supplementary Material

Additional file 1: Figure S1 Expression of OPN in primary NSCLC. Representative photomicrographs of NSCLC specimens stained with anti-OPN. Negative, weak and strong staining is demonstrated in A, B and C, respectively. Click here for file

## References

[B1] Jemal A Bray F Center MM Ferlay J Ward E Forman D Global cancer statistics CA Cancer J Clin 2011 61 69 90 10.3322/caac.20107 21296855

[B2] Ishikawa M Miyahara R Sonobe M Horiuchi M Mennju T Nakayama E Kobayashi M Kikuchi R Kitamura J Imamura N Higher expression of EphA2 and ephrin-A1 is related to favorable clinicopathological features in pathological stage I non-small cell lung carcinoma Lung Cancer 2012 76 431 438 10.1016/j.lungcan.2011.12.004 22236865

[B3] Goldstraw P Ball D Jett JR Le Chevalier T Lim E Nicholson AG Shepherd FA Non-small-cell lung cancer Lancet 2011 378 1727 1740 10.1016/S0140-6736(10)62101-0 21565398

[B4] Rud AK Lund-Iversen M Berge G Brustugun OT Solberg SK Maelandsmo GM Boye K Expression of S100A4, ephrin-A1 and osteopontin in non-small cell lung cancer BMC Cancer 2012, Aug 1 12 333 10.1186/1471-2407-12-333 22853000 PMC3458900

[B5] Boye K Maelandsmo GM S100A4 and metastasis: a small actor playing many roles Am J Pathol 2010 176 528 535 10.2353/ajpath.2010.090526 20019188 PMC2808059

[B6] Matsubara D Niki T Ishikawa S Goto A Ohara E Yokomizo T Heizmann CW Aburatani H Moriyama S Moriyama H Differential expression of S100A2 and S100A4 in lung adenocarcinomas: clinicopathological significance, relationship to p53 and identification of their target genes Cancer Sci 2005 96 844 857 10.1111/j.1349-7006.2005.00121.x 16367903 PMC11159992

[B7] Kimura K Endo Y Yonemura Y Heizmann CW Schafer BW Watanabe Y Sasaki T Clinical significance of S100A4 and E-cadherin-related adhesion molecules in non-small cell lung cancer Int J Oncol 2000 16 1125 1131 10811984 10.3892/ijo.16.6.1125

[B8] Tsuna M Kageyama S Fukuoka J Kitano H Doki Y Tezuka H Yasuda H Significance of S100A4 as a prognostic marker of lung squamous cell carcinoma Anticancer Res 2009 29 2547 2554 19596927

[B9] De Petris L Orre LM Kanter L Pernemalm M Koyi H Lewensohn R Lehtio J Tumor expression of S100A6 correlates with survival of patients with stage I non-small-cell lung cancer Lung Cancer 2009 63 410 417 10.1016/j.lungcan.2008.06.003 18620780

[B10] Feng J Zhang X Zhu H Wang X Ni S Huang J FoxQ1 overexpression influences poor prognosis in non-small cell lung cancer, associates with the phenomenon of EMT PLoS One 2012 7 6 e39937 doi: 10.1371/journal.pone.0039937. Epub 2012 Jun 28 10.1371/journal.pone.0039937 22761930 PMC3386178

[B11] Wu J Pungaliya P Kraynov E Bates B Identification and quantification of osteopontin splice variants in the plasma of lung cancer patients using immunoaffinity capture and targeted mass spectrometry Biomarkers 2012 17 125 133 10.3109/1354750X.2011.643485 22188260

[B12] Zhao B Sun T Meng F Qu A Li C Shen H Jin Y Li W Osteopontin as a potential biomarker of proliferation and invasiveness for lung cancer J Cancer Res Clin Oncol 2010 137 1061 1070 10.1007/s00432-010-0968-7 PMC11828203 21207061

[B13] Boldrini L Donati V Dell'Omodarme M Prati MC Faviana P Camacci T Lucchi M Mussi A Santoro M Basolo F Fontanini G Prognostic significance of osteopontin expression in early-stage non-small-cell lung cancer Br J Cancer 2005 93 453 457 10.1038/sj.bjc.6602715 16091764 PMC2361587

[B14] Donati V Boldrini L Dell'Omodarme M Prati MC Faviana P Camacci T Lucchi M Mussi A Santoro M Basolo F Fontanini G Osteopontin expression and prognostic significance in non-small cell lung cancer Clin Cancer Res 2005 11 6459 6465 10.1158/1078-0432.CCR-05-0541 16166420

[B15] Weber GF Lett GS Haubein NC Osteopontin is a marker for cancer aggressiveness and patient survival Br J Cancer 2010 103 861 869 10.1038/sj.bjc.6605834 20823889 PMC2966627

[B16] Hu Z Lin D Yuan J Xiao T Zhang H Sun W Han N Ma Y Di X Gao M Overexpression of osteopontin is associated with more aggressive phenotypes in human non-small cell lung cancer Clin Cancer Res 2005 11 4646 4652 10.1158/1078-0432.CCR-04-2013 16000556

[B17] Schneider S Yochim J Brabender J Uchida K Danenberg KD Metzger R Schneider PM Salonga D Holscher AH Danenberg PV Osteopontin but not osteonectin messenger RNA expression is a prognostic marker in curatively resected non-small cell lung cancer Clin Cancer Res 2004 10 1588 1596 10.1158/1078-0432.CCR-0565-3 15014008

[B18] Chang YS Kim HJ Chang J Ahn CM Kim SK Elevated circulating level of osteopontin is associated with advanced disease state of non-small cell lung cancer Lung Cancer 2007 57 373 380 10.1016/j.lungcan.2007.04.005 17513004

[B19] Isa S Kawaguchi T Teramukai S Minato K Ohsaki Y Shibata K Yonei T Hayashibara K Fukushima M Kawahara M Serum osteopontin levels are highly prognostic for survival in advanced non-small cell lung cancer: results from JMTO LC 0004 J Thorac Oncol 2009 4 1104 1110 10.1097/JTO.0b013e3181ae2844 19620934

[B20] Takenaka M Hanagiri T Shinohara S Yasuda M Chikaishi Y Oka S Shimokawa H Nagata Y Nakagawa M Uramoto H Serum level of osteopontin as a prognostic factor in patients who underwent surgical resection for non-small-cell lung cancer Clin Lung Cancer 2012 14 3 288 294 doi: 10.1016/j.cllc.2012.09.005. Epub 2012 Nov 1 23122494 10.1016/j.cllc.2012.09.005

[B21] Giacopelli F Marciano R Pistorio A Catarsi P Canini S Karsenty G Ravazzolo R Polymorphisms in the osteopontin promoter affect its transcriptional activity Physiol Genomics 2004 20 87 96 10.1152/physiolgenomics.00138.2004 15479859

[B22] Pasquale EB Eph receptors and ephrins in cancer: bidirectional signalling and beyond Nat Rev Cancer 2010 10 165 180 10.1038/nrc2806 20179713 PMC2921274

[B23] Saintigny P Peng S Zhang L Sen B Wistuba II Lippman SM Girard L Minna JD Heymach JV Johnson FM Global Evaluation of Eph receptors and ephrins in lung adenocarcinomas identifies EphA4 as an inhibitor of cell migration and invasion Mol Cancer Ther 2012 11 2021 2032 10.1158/1535-7163.MCT-12-0030 22807579 PMC3438283

[B24] Flatmark K Maelandsmo GM Mikalsen SO Nustad K Varaas T Rasmussen H Meling GI Fodstad O Paus E Immunofluorometric assay for the metastasis-related protein S100A4: release of S100A4 from normal blood cells prohibits the use of S100A4 as a tumor marker in plasma and serum Tumour Biol 2004 25 31 40 10.1159/000077721 15192310

[B25] Bjorheim J Gaudernack G Giercksky KE Ekstrom PO Direct identification of all oncogenic mutants in KRAS exon 1 by cycling temperature capillary electrophoresis Electrophoresis 2003 24 63 69 10.1002/elps.200390032 12652573

[B26] Blasberg JD Pass HI Goparaju CM Flores RM Lee S Donington JS Reduction of elevated plasma osteopontin levels with resection of non-small-cell lung cancer J Clin Oncol 2010 28 936 941 10.1200/JCO.2009.25.5711 20085934 PMC2834433

[B27] Sharma P Kumar S Kundu GC Transcriptional regulation of human osteopontin promoter by histone deacetylase inhibitor, trichostatin A in cervical cancer cells Mol Cancer 2010, Jul 7 9 178 doi: 10.1186/1476-4598-9-178 10.1186/1476-4598-9-178 20609221 PMC2911447

[B28] Shinohara ML Kim HJ Kim JH Garcia VA Cantor H Alternative translation of osteopontin generates intracellular and secreted isoforms that mediate distinct biological activities in dendritic cells Proc Natl Acad Sci USA 2008 105 7235 7239 10.1073/pnas.0802301105 18480255 PMC2438233

[B29] Lanteri P Lombardi G Colombini A Grasso D Banfi G Stability of osteopontin in plasma and serum Clin Chem Lab Med 2012 50 11 1979 1984 22718644 10.1515/cclm-2012-0177

[B30] Dong QZ Zhang XF Zhao Y Jia HL Zhou HJ Dai C Sun HJ Qin Y Zhang WD Ren N Osteopontin promoter polymorphisms at locus -443 significantly affect the metastasis and prognosis of human hepatocellular carcinoma Hepatology 2013 57 1024 1034 10.1002/hep.26103 23079960

[B31] Schultz J Lorenz P Ibrahim SM Kundt G Gross G Kunz M The functional -443 T/C osteopontin promoter polymorphism influences osteopontin gene expression in melanoma cells via binding of c-Myb transcription factor Mol Carcinog 2009 48 14 23 10.1002/mc.20452 18459127

[B32] Chen Y Liu H Wu W Li Y Li J Osteopontin genetic variants are associated with overall survival in advanced non-small-cell lung cancer patients and bone metastasis J Exp Clin Cancer Res 2013, Jul 24 32 45 doi: 10.1186/1756-9966-32-45 10.1186/1756-9966-32-45 23883434 PMC3728114

