# Supplementary figures and images for: Osteopontin is a prognostic biomarker in non-small cell lung cancer
Source: BMC Cancer. 2013 Nov 11;13:540. doi: 10.1186/1471-2407-13-540 (PMC3830440; doi:10.1186/1471-2407-13-540)

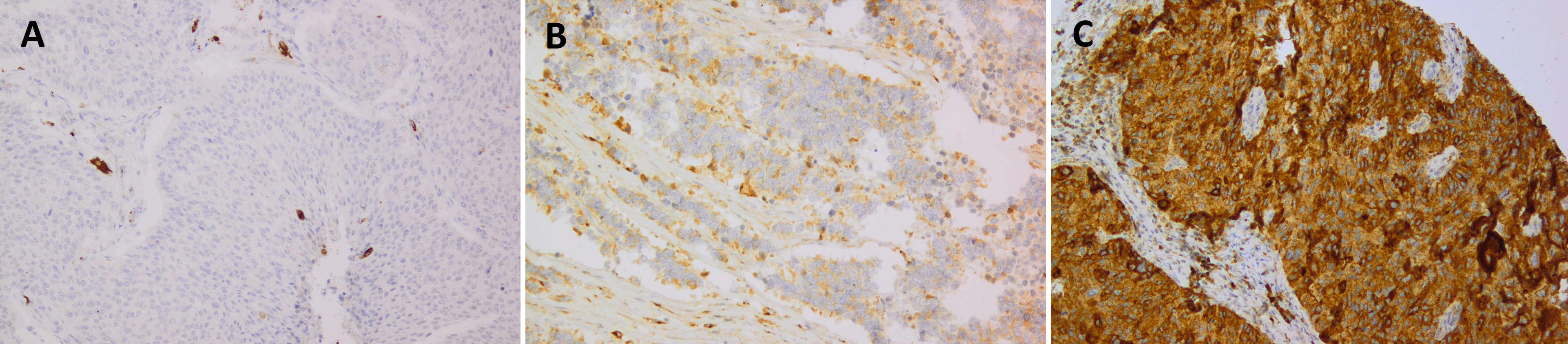

Supplement: Additional file 1: Figure S1 — Expression of OPN in primary NSCLC. Representative photomicrographs of NSCLC specimens stained with anti-OPN. Negative, weak and strong staining is demonstrated in A, B and C, respectively. [file 1471-2407-13-540-S1.jpeg]
